# Molecular Epidemiologic Source Tracking of Orally Transmitted Chagas Disease, Venezuela

**DOI:** 10.3201/eid1907.121576

**Published:** 2013-07

**Authors:** Maikell Segovia, Hernán J. Carrasco, Clara E. Martínez, Louisa A. Messenger, Anaibeth Nessi, Juan C. Londoño, Raul Espinosa, Cinda Martínez, Mijares Alfredo, Rafael Bonfante-Cabarcas, Michael D. Lewis, Belkisyolé A. de Noya, Michael A. Miles, Martin S. Llewellyn

**Affiliations:** Universidad Central de Venezuela Instituto de Medicina Tropical, Caracas, Venezuela (M. Segovia, H.J. Carrasco, C.E. Martínez, A. Nessi, B.A. de Noya);; London School of Hygiene and Tropical Medicine, London, UK (L.A. Messenger, M.D. Lewis, M.A. Miles, M.S. Llewellyn);; Universidad Central de Venezuela, Caracas (J.C. Londoño);; Instituto Venezolano de los Seguros Sociales, Caracas (R. Espinosa);; Instituto Venezolano de Investigaciones Científicas, Caracas (M. Alfredo);; Ministerio del Poder Popular para la Salud, Maracay, Estado Aragua, Venezuela (C. Martínez);; Universidad Centroccidental Lisandro Alvarado, Barquisimeto, Venezuela (R. Bonfante-Cabarcas)

**Keywords:** Trypanosoma cruzi, oral, orally transmitted, Chagas disease, Venezuela, Caracas, Vargas State, molecular epidemiology, parasites, trypanosome, zoonoses, vector-borne infections

## Abstract

Oral outbreaks of Chagas disease are increasingly reported in Latin America. The transitory presence of *Trypanosoma cruzi* parasites within contaminated foods, and the rapid consumption of those foods, precludes precise identification of outbreak origin. We report source attribution for 2 peri-urban oral outbreaks of Chagas disease in Venezuela via high resolution microsatellite typing.

Rapid urbanization presents new challenges for Chagas disease control in Latin America. Foci of disease are now reported in slums surrounding several Andean cities (*1*–*3*). Oral transmission is believed responsible for recent outbreaks of Chagas disease, most of which were characterized by atypically severe symptoms (*4*,*5*). Many cases have occurred in urban settings (*5*,*6*), amplifying the size and effect of the outbreaks.

Sources of orally transmitted disease outbreaks vary, but contaminated food and juices are often blamed. However, after a contaminated food is eaten, it may take weeks for the onset of clinical signs and symptoms, and direct molecular and cytological incrimination of a particular batch of food/beverage has not been possible (*5*). Thus, evidence pointing to particular foodstuffs is often circumstantial.

Molecular epidemiologic analyses of human and environmental isolates are routinely used to track the source of outbreaks caused by foodborne pathogens. High-resolution molecular markers have been developed and validated for *Trypanosoma cruzi*, the parasite that causes Chagas disease (*7*,*8*). These markers, used in conjunction with careful sampling, can identify the source of foodborne outbreaks.

## The Study

We studied 2 outbreaks of orally transmitted Chagas disease (120 cases, 5 deaths). The first occurred in Chichiriviche, Vargas State, a coastal community (population ≈800 persons) ≈50 km northwest of Caracas, Venezuela. The outbreak occurred at a primary school where food was prepared on site. In early April 2009, a total of 71 children (6–13 years of age) who attended the morning school shift and 14 adults became ill. Exposure of these persons to *T. cruzi* was established by use of IgM and IgG ELISA. Parasitemia was observed in 33 of the patients with serologic results positive for *T. cruzi* infection (*9*,*10*). 

The second outbreak occurred in Antimano, a peri-urban slum southwest of central Caracas (Technical Appendix Figure 1). In May 2010, 35 patients with suspected *T. cruzi* infection were examined at Hospital Miguel Perez Carreno in Caracas. Patients reported that they regularly ate at the same local communal canteen. Among the patients tested, 15 were positive for *T. cruzi* IgM and IgG (*9*). Parasitemia in 14 patients was confirmed indirectly by hemoculture. Of the 35 patients, 21 (2 adults, 19 children) were hospitalized. 

To enable outbreak source attribution, we undertook intensive additional sampling of contemporary, nonhuman sources local to each outbreak and of human and nonhuman sources from more distant localities throughout Venezuela. In total, 246 *T. cruzi* strains and clones were typed for 23 microsatellite markers (Technical Appendix 1 Table) (*8*). A list of the samples and their sites of origin is in Technical Appendix 2).

**Figure 1:**
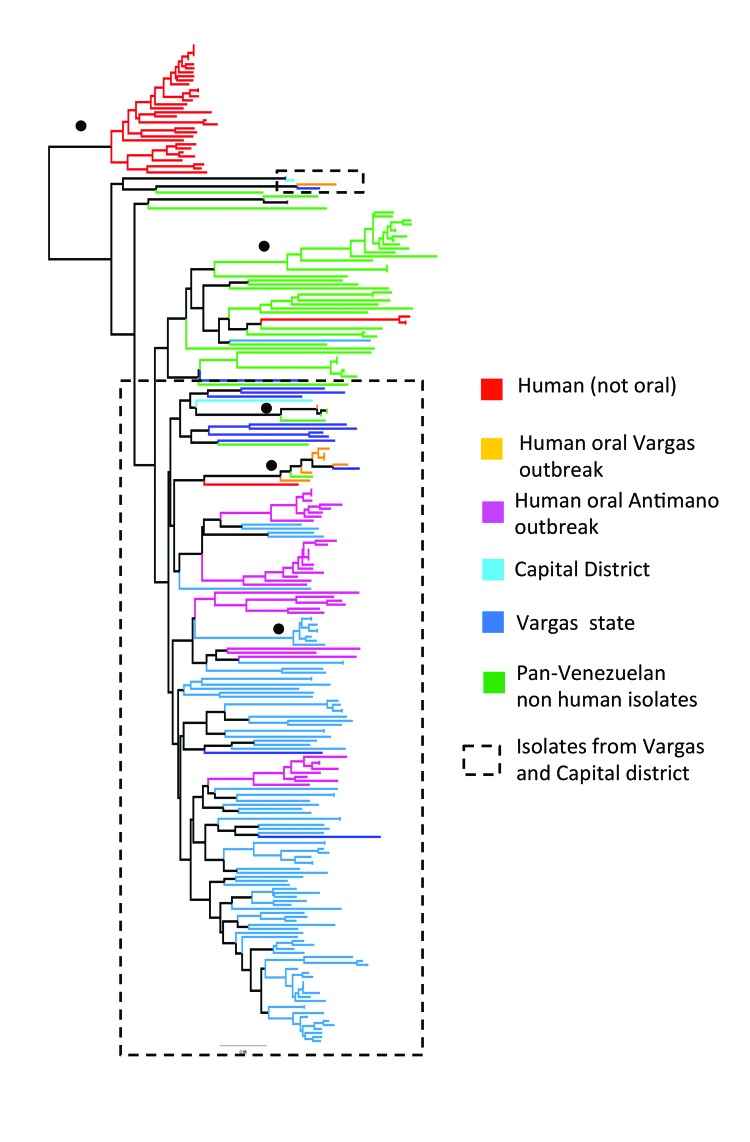
Unrooted neighbor joining tree showing genetic clustering among *Trypanosoma cruzi* isolates from 2 outbreaks of oral disease in northern Venezuela. Based on pairwise genetic distances (1 – proportion of shared alleles) between multilocus microsatellite profiles (23 loci) generated from 246 isolates and clones. Black circles indicate nodes with >60% bootstrap support. Branch color key is shown. Dashed boxes indicate isolates associated with the outbreaks.

**Figure 2:**
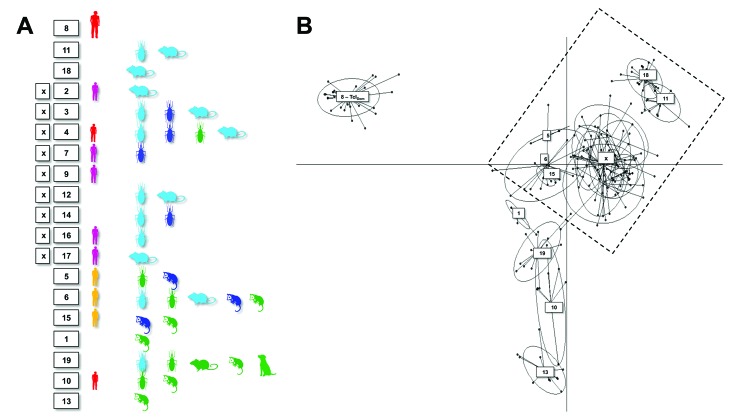
Discriminant analysis of principal components showing genetic clustering among *Trypanosoma cruzi* isolates from 2 outbreaks of oral disease in northern Venezuela. Six principal components were retained, explaining 80% of the diversity. Ellipses correspond to the optimal (as defined by the Bayesian information criterion minimum) number of population clusters among the genotypes analyzed. Images indicate sample host origin (human, rodent, marsupial, or triatomine), while colors correspond to the key in Figure 1. A full list of samples and population assignments (numbered boxes) is included in Technical Appendix 2. Dashed box indicates the isolates associated with the outbreaks.

Individual level sample clustering was defined first by constructing a neighbor joining tree based on pairwise distances between multilocus genotypes (Figure 1). A second analysis used *K*-means clustering and discriminant analysis of principal components (Figure 2) (*11*). To assess connectivity between human and nonhuman outbreak cases, pairwise genetic differentiation (*F*_ST_) was calculated (Table 1). Population-level genetic diversity was assessed first by calculating allelic richness then private allele frequency over loci between each human–nonhuman population pair (Table 2). Geographic sampling distribution is shown in Technical Appendix 1 Figure 2.

**Table 1:** *F*_ST_ values in a 4-way comparison for differentiation between *Trypanosoma cruzi* isolates derived from humans and the local environment during an outbreak of orally transmitted Chagas disease in 2 areas of Venezuela*

Isolate, location	Human isolates from		Nonhuman isolates from	
	Antimano	Chichiriviche	Antimano	Chichiriviche
Human				
Antimano		0.000	0.000	0.000
Chichiriviche	0.201		0.000	0.004
Nonhuman				
Antimano	0.093	0.170		0.000
Chichiriviche	0.088	0.053	0.079	

*Lower left shows linearized *F*_ST_ (genetic differentiation) values; upper right shows associated p values.

**Table 2:** Sample size corrected diversity between *Trypanosoma cruzi* isolates derived from humans and the local environment during an outbreak of orally transmitted Chagas disease in 2 areas of Venezuela*

Isolate, location	No. isolates/no. genotypes	Sample size corrected allelic richness ± SE	Mean no. private alleles/locus ± SE
Human			
Antimano	30/26	2.735 ± 0.291	0.32 ± 0.113
Chichiriviche	12/9	3.459 ± 0.412	0
Nonhuman			
Antimano	107/91	2.946 ± 0.320	0.86 ± 0.203
Chichiriviche	13/13	3.443 ± 0.409	0

Clustering results determined by discriminant analysis of principal components and neighbor joining were broadly congruent. In the former, 19 clusters were defined; sample allocations are included in Technical Appendix 2. Substantial overlap existed between some clusters, especially those from Capital and Vargas States (e.g., those labeled “x” in Figure 2), while others were highly distinct (e.g., cluster 8 in Figure 2). Human isolates from both oral outbreaks are extremely distinct from non-orally transmitted isolates collected from humans throughout Venezuela. Almost all these presumably vector-transmitted strains are closely related to one another, despite their geographic dispersal (cluster 8 in Figure 2). By comparison, oral outbreak strains that were isolated a mere 50 km apart (clusters 2, 5, 7, 9, 15–17) are far more globally diverse. Unlike most human isolates in Venezuela, which are distinct from nonhuman strains, oral outbreak isolates are interspersed among nonhuman strains from Venezuela. Furthermore, samples from both outbreaks clustered among nonhuman strains local to that outbreak, clearly indicating a local origin. Oral samples from each outbreak are polyphyletic with respect to strains from their immediate environment, a finding consistent with multiple contamination events or multiclonal infection sources.

*F*_ST_ values further support connectivity between outbreak and local environmental samples in both Antimano and Chichiriviche (Table 1). A lack of private alleles between human and nonhuman isolates also supports a local source for the Chichiriviche outbreak (Table 2). *F*_ST_ values in the 4-way comparison between outbreak and local environmental strains are, however, somewhat equivocal with respect to the entire dataset (Table 1). Cluster analysis showed that the human and nonhuman strains from Chichiriviche interspersed with strains from other states in Venezuela (Figure 2). Thus, we cannot confirm a uniquely local origin for the Chichiriviche outbreak, despite a low value for *F*_ST_, and it is possible that some contaminating strains originated elsewhere.

## Conclusions

This study demonstrates the value of rigorous molecular epidemiologic analysis of orally transmitted *T. cruzi* outbreaks, including the importance of appropriate sampling to identify the origin of the infecting strains. The foodstuff that propagated the peri-urban outbreak in Antimano was certainly contaminated locally. An active nonhuman transmission cycle in the slums of Caracas, maintained by *Rattus rattus* rodents and *Panstrongylus geniculatus* triatomines, is the likely source. The Chichiriviche outbreak, however, has potential sources both in and outside the immediate area. As found in Chagas disease outbreaks linked to açaí palm fruit in Brazil (*12*), the *T. cruzi* parasite can survive for several days in some foodstuffs (*13*). Also, triatomines can survive for months in harvested crops; thus, multiple hygiene interventions are potentially necessary along the food production line (*14*). Nonetheless, if the foodstuff implicated was prepared locally, local contamination represents the most likely source of the outbreak. Study of additional nonhuman strains from Chichiriviche is necessary to support this assertion.

Crucial to understanding parasite transmission in general, we believe, are genetic differences between strains from orally and non-orally transmitted human cases. All TcI strains appear to be infective to humans and adapted to long-term carriage (*8*). However, the presence of a common, reduced-diversity TcI genotype cluster (TcI_DOM_) among a high proportion of human Chagas disease cases in South America is also well established (*7*,*8*). We originally hypothesized that TcI_DOM_ was maintained, despite the presence of sympatric and infective sylvatic strains, because of low parasite transmission efficiency by invasive sylvatic vectors (*8*). Oral transmission is likely to be much more efficient. Thus, unlike TcI_DOM_ strains, those from orally transmitted *T. cruzi* cases demonstrated high genetic diversity and clearly originated from local nonhuman *T. cruzi* populations. However, it is also true that all TcI_DOM_ strains we isolated originated from patients with chronic infection, and all orally transmitted cases were in the acute phase. We cannot, therefore, rule out a role for immune selection in driving the frequency of TcI_DOM_ infections among humans; such selection represents an intriguing topic for future enquiry.

Molecular tools and reference datasets are now available to determine the source of acute Chagas disease outbreaks within days of their occurrence. The plummeting cost of such analyses means it is time to apply population genetic techniques and markers developed for trypanosomes as genuine epidemiologic tools.

Technical Appendix 1 Photo of Antimano, a suburban slum of Caracas, map showing the geographic distribution of *Trypanosoma cruzi* I isolates analyzed, and primers and chromosomal positions for microsatellite loci used in a study of the molecular epidemiologic source tracking of 2 outbreaks of oral Chagas disease, Venezuela.

Technical Appendix 2 Trypanosoma cruzi isolates and clones analyzed in a study of the molecular epidemiologic source tracking of 2 outbreaks of oral Chagas disease, Venezuela.
