# Supplementary material for: Molecular Epidemiologic Source Tracking of Orally Transmitted Chagas Disease, Venezuela
Source: Emerg Infect Dis. 2013 Jul;19(7):1098–101. doi: 10.3201/eid1907.121576 (PMC3903455; doi:10.3201/eid1907.121576)
Supplement: Technical Appendix 1 — Photo of Antimano, a suburban slum of Caracas, map showing the geographic distribution of Trypanosoma cruzi I isolates analyzed, and primers and chromosomal positions for microsatellite loci used in a study of the molecular epidemiologic source tracking of 2 outbreaks of oral Chagas disease, Venezuela. [file 12-1576-Techapp-s1.pdf]

Article DOI: http://dx.doi.org/10.3201/eid1907.121576

Molecular Epidemiologic Tracking of Source of Orally Transmitted Chagas Disease, Venezuela

Technical Appendix 1

Technical Appendix Table. Primers and chromosomal positions for microsatellite loci used in a study of the molecular epidemiologic source tracking of 2 outbreaks of oral Chagas disease, Venezuela.

Chromosome	Primer code	Repeat type	Forward/Reverse Primer (5'-3')
6	6529(CA)a	$(CA)_n$	TGTGAAATGATTTGACCCGA<br>AGAGTCACGCCGCAAAGTAT
6	6529(TA)b	$(TA)_n$	TGAAGGAGATTCTCTGCGGT<br>CTCTCATCTTTTGTTGTGTCCG
6	mclf10	$(CA)_nA(CA)_n$	GCGTAGCGATTCATTTCC<br>ATCCGCTACCACTATCCAC
10	6855(TA)(GA)	$(TA)_n(GA)_n$	TGTGATCAACGCGCATAAAT<br>TTCCATTGCCTCGTTTTAGA
19	TcUn3	Unknown	CTTAAAGAGATACAAGAGGGAAGG<br>CTGTTATTTCAATAACACGGGG
19	10101(TA)	$(TA)_n$	AACCCGCGCAGATACATTAG<br>TTCATTTGCAGCAACACACA
27	10101(TC)	$(TC)_n$	CGTACGACGTGGACACAAAC<br>ACAAGTGGGTGAGCCAAAAG
27	10101(CA)a	$(CA)_n$	GTCGCCATCATGTACAAACG<br>CTGTTGGCGAATGGTCATAA
34	6559(TC)	$(TC)_n$	CGCTCTCAAAGGCACCTTAC<br>ATATGGACGCGTAGGAGTGC
37	10187(TTA)	$(TTA)_n$	GAGAGAGATTCGGAAACTAATAGC<br>CATGTCCCTTCCTCCGTAAA
37	10187(CA)(TA)	$(CA)_n(TA)_n$	CATGTCATTAAGTGGCCACG<br>GCACATGTTGGTTGTTGGAA
37	10187(TA)	$(TA)_n$	AGAAAAAGGTTTACAACGAGCG<br>CGATGGAGAACGTGAAACAA
37	10187(GA)	$(GA)_n$	GTCACACCACTAGCGATGACA<br>ACTGCACAATACCCCCTTTG
37	TcUn2	Unknown	AACAAAATCTAGCGTCTACCATCC<br>GGTGTTGGCGTGTATGATTG
37	TcUn4	Unknown	ATGCTCCGCAACATATTACTCA<br>GTCGAGCTTCTGTTGTTCCC
39	6925(TG)b	$(TG)_n$	GAAACGCACTCACCCACAC<br>GGTAGCAACGCCAAACTTTC
39	7093(TC)	$(TC)_n$	CCAACATTCAACAAGGGAAA<br>GCATGAATATTGCCGGATCT
39	6925(CT)	$(CT)_n$	CATCAAGGAAAAACGGAGGA<br>CGGTACCACCTCAAGGAAAG
39	7093(TA)c	$(TA)_n$	CGTGTGCACAGGAGAGAAAA<br>CGTTTGGAGGAGGATTGAGA
39	7093(TCC)	$(TCC)_n$	AGACGTTCATATTCGCAGCC<br>AGCCACATCCACATTTCCTC
40	11283(TCG)	$(TCG)_n$	ACCACCAGGAGGACATGAAG<br>TGTACACGGAACAGCGAAG
40	11283(TA)b	$(TA)_n$	AACATCCTCCACCTCACAGG<br>TTTGAATGCGAGGTGGTACA
41	10359(CA)(GA)	$(CA)_n(GA)_n$	AGTCCTACTGCCTCCTTGCA<br>CTGTTGGCGAATGGTCATAA

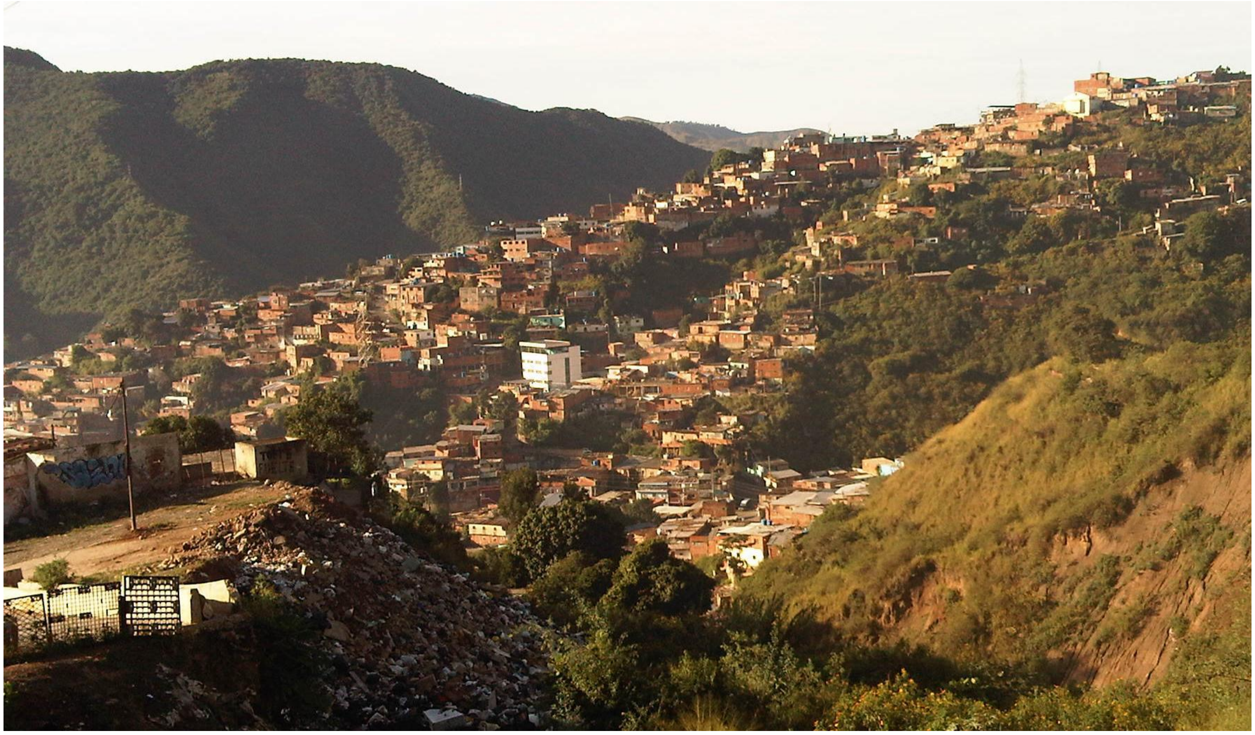

Technical Appendix Figure 1. Photo of Antimano, a suburban slum of Caracas, Venezuela.

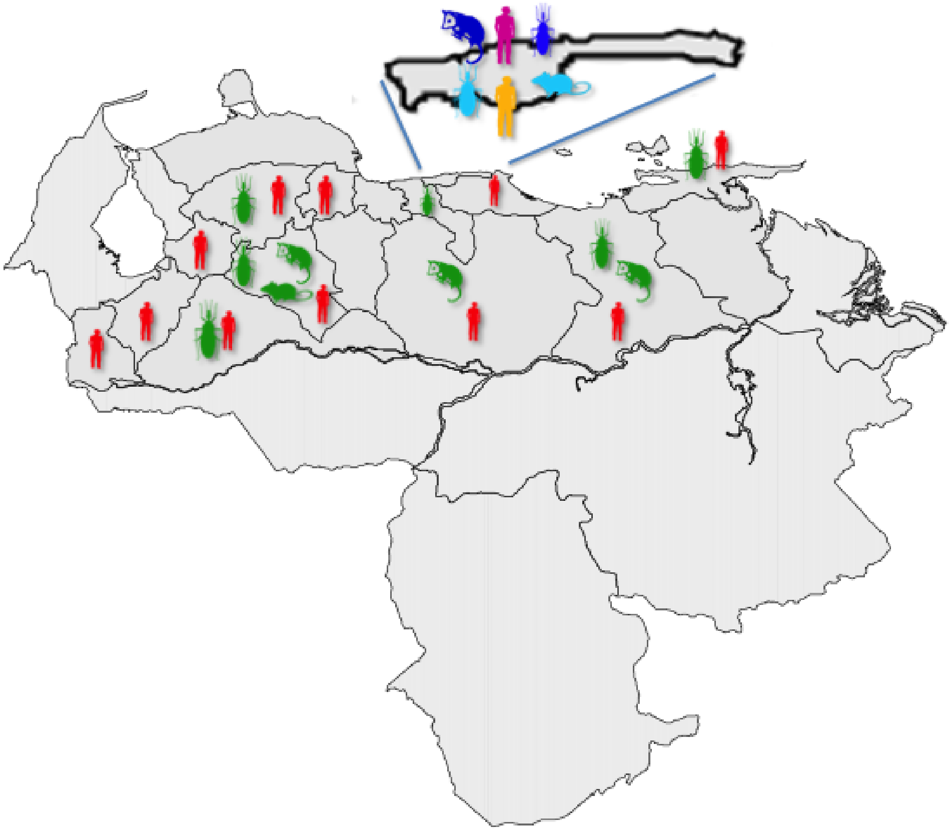

Technical Appendix Figure 2. Geographic distribution of *Trypanosoma cruzi* isolates of the TcI genetic lineage included in a study of the molecular epidemiologic tracking of the source 2 Chagas disease outbreaks in Venezuela. Images indicate host origin (human, rodent, marsupial, or triatomine) of the samples; colors denote genetic population as explained in text Figure 2. Inset shows the capital city (Caracas) and surrounding Vargas State, where outbreaks of oral Chagas disease occurred in the cities of Antimano and Chichiriviche, respectively.
